# Regional Socioeconomic Deprivation in Germany and Adherence to the 24-h Movement Guidelines among Children and Adolescents

**DOI:** 10.3390/children10081392

**Published:** 2023-08-15

**Authors:** Vivien Suchert, Reiner Hanewinkel, Clemens Neumann, Julia Hansen

**Affiliations:** Institute for Therapy and Health Research, IFT-Nord, Harmsstraße 2, 24114 Kiel, Germany; suchert@ift-nord.de (V.S.); neumann@ift-nord.de (C.N.); hansen@ift-nord.de (J.H.)

**Keywords:** inequality, observational study, social gradient, screen time, sleep, moderate to vigorous physical activity, young people

## Abstract

To examine the relationship between regional socioeconomic deprivation and adherence to the 24-h movement guidelines among children and adolescents, a cross-sectional study was conducted. A total of 17,433 students aged 9–17 participated in a survey in winter 2021/2022. Daily screen time (ST), moderate-to-vigorous physical activity (MVPA), and sleep behavior were outcome variables. The German Index of Socioeconomic Deprivation (GISD), a regional socioeconomic objective measure, was the exposure variable. Associations between GISD and health behaviors were examined using regression models. Models were adjusted for age, gender, school type, and individual self-reported socioeconomic status (SES). The proportions of meeting the MVPA, ST, and sleep duration guidelines were 14%, 22%, and 34%, respectively. A total of 2.3% met all guidelines. Students from the most deprived communities were half as likely to meet all three guidelines compared to students from the most affluent regions (OR = 0.49 [0.28; −0.03], *p* = 0.010). There was a consistent relationship between GISD and lower levels of adherence to screen time guidelines (most deprived compared to most affluent: OR = 0.49 [0.38; 0.64], *p* < 0.001). There was no association between GISD and adherence to sleep time guidelines. We found mixed results for the association between GISD and MVPA. Regional SES appears to be an important factor associated with screen time. Screen time should be limited through intervention programs, especially in disadvantaged areas. Parents should be made aware of their children’s increased media consumption. Recommendations for screen time should be clearly communicated, as should the health disadvantages of increased media consumption in childhood and adolescence.

## 1. Introduction

Screen time, sleep duration, and physical activity are important components of a healthy lifestyle for children and adolescents [1]. Regular physical activity (PA) is linked to several positive health outcomes, including improved cardiovascular health, better mental health, and a reduced risk of chronic disease [2]. Screen time (ST) refers to the amount of time spent engaging in screen-based activities, such as watching television, playing video games, or using electronic devices like smartphones or tablets. Excessive screen time has been linked to negative health outcomes in children and adolescents, including obesity, poor mental health, and reduced academic performance [3,4]. Sleep is a critical component of health and well-being, especially during childhood and adolescence when growth and development are rapid. However, poor sleep habits are common among young people, and insufficient sleep is associated with a number of negative outcomes, including poor academic performance, obesity, and mental health problems [5]. Because all three behaviors (physical activity, screen time, sleep duration) are interrelated, several countries have followed Canada in adopting 24-h movement guidelines for children and adolescents [6].

Recommendations suggest 9 to 11 h of sleep per night for children (ages 5–13 years) and 8 to 10 h for adolescents (ages 13–17 years) [7]. Both groups should accumulate at least 1 h of moderate-to-vigorous physical activity (MVPA) and limit ST (i.e., exposure to all screen-based digital media) to 2 h or less within a 24-h period (World Health Organization, 2020 [8]). Meeting recommendations for all three behaviors may have a greater association with health outcomes than meeting any one recommendation in isolation [9]. However, a recent meta-analysis found that overall only 10.3% of children and 2.7% of adolescents adhere to all three guidelines, while 15.6% of children and 28.6% of adolescents meet none of the three movement recommendations [6]. With more than 80% of students aged 11 to 17 not meeting physical activity guidelines [10], MVPA appears to be the most important aspect of the problem. Furthermore, several studies have found that most older children and adolescents exceed screen time recommendations [9,11,12].

According to the social–ecological model, health behavior is influenced by a collection of subsystems that occur at different levels (individual, interpersonal, institutional, community, and public policy) [13]. According to this approach, socioeconomic and environmental factors can influence health behavior, and it has already been shown that socioeconomic status (SES) is widely considered to be a fundamental determinant of health [14]. The association between poor health and low SES, also known as the social gradient or social inequalities in health, is well documented [15,16]. The association extends throughout the lifespan and affects all population groups [17]. SES can be measured at the individual/family level, for example, when an individual assesses their own SES, which allows them to assess their own material resources and determine where they place them in relation to their community [18]. However, SES can also be determined at the regional or area level. Regional or environmental socioeconomic disparities are also referred to in the literature as neighborhood SES, community- or area-level SES, or regional SES and are based on objective factors such as median household income in a given region. Regional SES is associated with poorer mental and physical health among young people [19,20].

Several studies have investigated the relationship between area-level SES and health behaviors in children and adolescents. In summary, regional socioeconomic disparities have been identified as a potential factor influencing ST and sleep, with low-SES communities often having higher levels of ST and sleep problems or lower sleep duration [21,22,23]. The association between area-level SES and PA in youth is controversial [21,24].

To date, the extent to which regional socioeconomic differences in Germany are associated with PA, ST, sleep, and adherence to the 24-hour movement guidelines in young people is not well documented. Previous evidence suggests that young people living in deprived areas in Germany are more likely to report higher ST and more PA, in contrast to many other studies that have found no or inverse associations with PA [21]. This paper aims to fill this gap by examining the association between regional socioeconomic deprivation and MVPA, ST, sleep behavior, and adherence to the 24-h movement guidelines alone or in combination in a large sample of German children and adolescents. In addition, we will investigate whether regional SES is linked to movement behaviors when controlling for other aspects of SES at a more individual level.

## 2. Materials and Methods

### 2.1. Study Design and Setting

Cross-sectional data were obtained from the sixth wave of the Präventionsradar, a school-based observational study conducted yearly in secondary schools in Germany since the 2016/2017 school year [25]. The survey was conducted between November 2021 and February 2022 in 13 states that gave permission to conduct the study (this includes all states except Hamburg, Bavaria, and Saarland).

All German secondary schools in the 13 states were invited by email to participate in the study. Once a school registered on the study’s website, it could enroll all classes from grades 5 to 10 (usually 10 to 16 years old). Any student in a registered class was eligible to participate. Informed written consent was obtained from parents before the survey was administered. Verbal consent was obtained from students on the day of data collection. Participation was strictly voluntary. All participants were allowed to withdraw from the study at any time without penalty. This included their participation in the survey before and during testing as well as the deletion of the collected data afterwards. Deletion was accomplished using an individual 7-digit code.

The study was approved by the state ministries of cultural affairs. Ethical approval was obtained from the German Psychological Society (RH 042015_1) on 15 June 2016.

### 2.2. Participants and Procedure

Data collection via a web-based questionnaire took place from November 2021 to February 2022. Instructed school staff supervised the survey at a time of their choosing. One class period (45 min) was usually required for participation. A total of 17,433 students aged 9–17 years participated in the survey. Dropout during the assessment for various reasons (i.e., withdrawal, skipping questions) resulted in a total N = 16,354 children and adolescents answering the question on PA (N = 17,120 answering the ST questions, and N = 16,317 answering the sleep questions).

### 2.3. Measures

#### 2.3.1. Exposure Variable

Regional socioeconomic deprivation: The German Index of Socioeconomic Deprivation (GISD) is a composite index of regional socioeconomic indicators in the areas of income, education, and occupation and was developed by the Robert Koch Institute [26]. Based on administrative data on education, employment, and income situations at the district and municipality level provided by the Federal Institute for Building Urban, and Spatial Research (Federal Institute for Building Urban and Spatial Research 2023), the GISD measures the level of socioeconomic deprivation. Approximately 600 indicators allow for urban-rural comparisons. The indicators used for the GISD are: share of employees without professional qualification, share of employees with a university degree, employment-to-population ratio, gross wages and salaries, net household income, share of school dropouts, private debtors per 100 inhabitants, and income tax revenue per capita. GISD scores are available for each region as a measure of socioeconomic deprivation (values between 0 (most affluent) to 1 (most deprived)). In addition, the units of the aforementioned regional levels were divided into five groups of twenty percent each (quintiles), with the bottom fifth representing “low” and the top fifth representing “high” socioeconomic deprivation.

We ranked the regional deprivation of the school community based on the quintiles of the GISD.

#### 2.3.2. Outcome Variables

Screen time: ST was assessed by asking, “On a typical school day, how much time do you spend in front of the computer, cell phone, or game console? This means all time, such as gaming, chatting, watching videos or movies, and more. Please enter the time in the boxes. Enter the time in hours and minutes.” Likewise, ST was assessed for weekends. Time on weekdays was weighted with 5 and on weekend days with 2, summed up, and divided by 7 to determine an average time per day. Children and adolescents who spent 2 h or less on ST met the ST guidelines.

Physical activity: MVPA levels were assessed using a validated two-item screening instrument [27]. Students were asked how many days in a normal week and in the past week they engaged in MVPA for at least 60 min. Responses could be given on an 8-point scale ranging from 0 days to 7 days. An average score was used to determine levels of MVPA. PA guidelines were coded with 7 days of MVPA as meeting PA guidelines and less than 7 days as not meeting PA guidelines.

Sleep: To measure sleep duration, participants were asked about their usual bedtime and wake-up time on weekdays. For analyses, sleep duration was rounded to whole numbers. In addition, meeting sleep guidelines was defined as 9–11 h per night (5–13 years) and 8–10 h per night (14–17 years) [7]. Sleep problems were assessed as follows: “The following question refers to the last six months: Have you had trouble falling asleep, or did you wake up more often?” Response options were “Never or hardly ever”, “Every month”, “Every week”, “Several times a week”, and “(Almost) daily”.

#### 2.3.3. Covariates

We assessed age as a continuous variable, gender (binary coded in 0 = female, 1 = male), and school type (0 = others, 1 = Gymnasium). The gymnasium is the most advanced type of secondary school in Germany that strongly emphasizes academic learning. Perceived social status was measured with the MacArthur Scale, which has been shown to have medium correlations with single objective SES indicators [28].

### 2.4. Statistical Analyses

Data analysis was performed using STATA, Version 17.0. First, we reported the prevalence of meeting PA, ST, and sleep guidelines. Second, unadjusted and adjusted multilevel logistic regression models were set up to examine the associations between regional socioeconomic deprivation and meeting PA, ST, sleep, and all three guidelines in combination. Because of the nested nature of the data, we included students’ school class as a level 2 variable in the models. Adjusted models controlled for the effect of age (continuous variable), gender (dichotomous variable), school type (dichotomous variable), and perceived social status (continuous variable) on the dependent variables. Third, multilevel linear regression models were constructed to examine the association between regional socioeconomic deprivation and MVPA, sleep, and ST. Fourth, we tested the robustness of the results. Instead of the five-level GISD variable, which we preferred for presentation and interpretation reasons, we used the metric GISD scale. In addition, we used linear regression instead of logistic regression in the model to test the association between GISD and adherence to all guidelines.

Data were weighted to census data to adjust for biases in representativeness. The weighting factor was created using data from the Federal Statistical Office and considers age, gender, and school type. Missing data were handled by listwise deletion.

## 3. Results

### 3.1. Sample Description

The total sample consisted of N = 17,433 students, with 49.8% female students. Age ranged from 9 to 17 years, with a mean age of 13.1 years (SD = 1.7 years). The mean perceived social status was 7.1 (SD = 1.5), and 60.4% of the sample attended Gymnasiums. There were 14.0%, 22.0%, and 34.2% of students meeting PA, ST, and sleep time guidelines, respectively. Nearly half of the students met none of the guidelines (48.7%), a third (35.0%) met one, 13.9% met two, and 2.3% met all guidelines. The distribution across GISD quintiles was as follows: quintile 1 (most affluent) 5.6%, quintile 2 23.5%, quintile 3 13.9%, quintile 4 35.3%, quintile 5 (most deprived) 21.8%. There were 42.6% of students who had no sleep problems; 18.4% reported sleep problems every month, 12.1% every week, 12.3% several times a week, and 14.7% daily.

Detailed sample characteristics for the total sample and adherence to each 24-h movement guideline are presented in Table 1.

Students who met the ST guidelines were more likely to be female (*p* < 0.001), more likely to attend Gymnasiums (*p* < 0.001), younger (*p* < 0.001), and had a higher perceived social status (*p* < 0.001) compared to students who did not meet the ST guidelines. Students who met the PA guidelines were more likely to be male (*p* < 0.001), more likely to attend school types other than a Gymnasium (*p* < 0.001), younger (*p* < 0.001), and had a higher perceived social status (*p* < 0.001) than students who did not meet the guidelines. Students who met the sleep time guidelines were more likely to be male (*p* < 0.001), younger (*p* < 0.001), and have a higher perceived social status (*p* < 0.001) than those who did not meet the guidelines. Students who met all three guidelines were more likely to be male (*p* < 0.001), younger (*p* < 0.001), and have a higher perceived social status (*p* < 0.001) compared to students who did not meet the three guidelines.

### 3.2. Association between Regional Socioeconomic Deprivation and Adherence to 24-h Movement Guidelines

There were no associations between regional socioeconomic deprivation and adherence to PA guidelines, as shown in Table 2. We found a consistent association between regional socioeconomic deprivation and adherence to ST guidelines in the way that higher deprivation was connected to lower levels of adherence to ST guidelines. In the adjusted model, the odds ratios of meeting screen time guidelines were significantly lower in the third (OR = 0.64 [0.48; 0.84], *p* = 0.001), fourth (OR = 0.54 [0.42; 0.69], *p* < 0.001), and fifth quintile (OR = 0.49 [0.38; 0.64], *p* < 0.001) than in the first quintile. There was no association between regional socioeconomic deprivation and adherence to sleep duration guidelines. We found a significant association between regional socioeconomic deprivation and adherence to all guidelines, such that higher deprivation was associated with lower odds of adherence. Compared with the most affluent quintile 1, the most deprived quintile 5 had half the odds of meeting all three guidelines (OR = 0.49 [0.28; −0.03], *p* = 0.010). Thus, students from more affluent SES communities were more likely to practice healthy sleep duration, limit screen time, and participate in physical activity. Specifically, this corresponds to 1.7% adherence for children and adolescents from the most deprived areas and 3.4% in the most affluent areas.

### 3.3. Association between Regional Socioeconomic Deprivation and PA, ST, and Sleep Behavior

As shown in Table 3, there was no association between regional socioeconomic deprivation and MVPA after adjusting for age, gender, school type, and perceived social status. Although not significant, the difference between students from the most deprived communities (quintile 5) and the most affluent communities (quintile 1) in terms of days with 60 min or more of MVPA was the largest (b = −0.15 [−0.34; 0.04], *p* = 0.131). While children and adolescents from the most disadvantaged regions (quintile 5) spent an average of 3.59 days per week engaging in the recommended daily amount of MVPA, children, and adolescents from the most affluent regions (quintile 1) spent an average of 3.74 days.

Sleep duration differed significantly between quintile 1 and quintile 5, with 7 h and 59 min in the most affluent regions and 8 h and 7 min in the most deprived regions. The difference was thus 8 min. There were consistent associations between sleep problems and socioeconomic deprivation. Students from the third quintile (b = 0.13 [0.01–0.25], *p* = 0.034), fourth quintile (b = 0.14 [0.04–0.24], *p* = 0.005), and fifth quintile (b = 0.19 [0.08–0.29], *p* < 0.001) were more likely to report sleep problems than children and adolescents from the most affluent quintile 1. Differences in the frequency of sleep problems between quintiles were found to be relatively small, with a mean scale score of 2.24 [95% 2.15–2.33] in the most affluent region, corresponding to sleep problems “once a month” versus a scale score of 2.43 [2.37–2.49] in the most deprived region (a scale score of 2.5 would correspond to sleep problems with a frequency of “once a week”).

**Figure 1:**
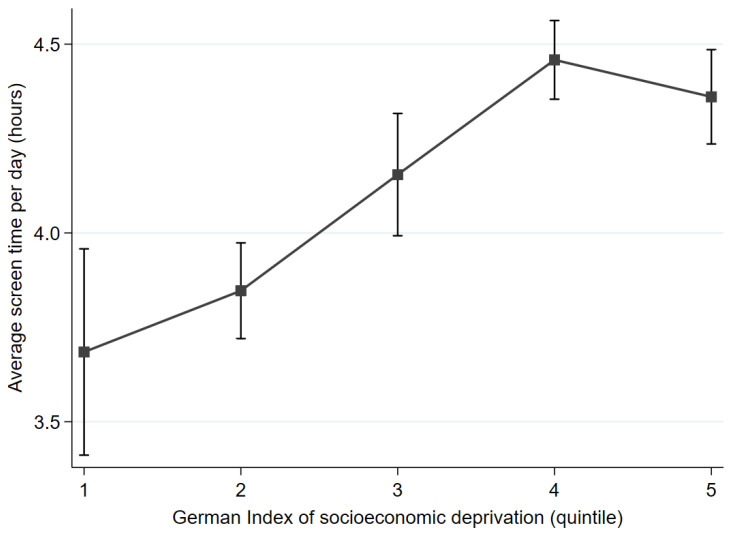
Association between screen time and quintiles of the German Index of Socioeconomic Deprivation, adjusted for age, gender, school type, and perceived social status.

There was a relationship between socioeconomic deprivation and ST in the way that ST was lower in affluent regions (GISD quintile 1) than in more deprived regions (quintile 4 b = 0.69 [0.48–0.90], *p* < 0.001; quintile 5 b = 0.61 [0.40–0.83], *p* < 0.001). While children and adolescents from GISD quintile 1 spent an average of 3 h and 47 min per day in front of screens, those who lived in disadvantaged areas had an average of 42 min more ST (4 h and 29 min per day). Figure 1 shows the association between ST and GISD quintiles after adjusting for age, gender, school type, and perceived social status.

### 3.4. Sensitivity Analysis

To test the robustness of the results and to verify that the results were not driven by our prioritized model specification, we reran the regression models with GISD as the metric predictor variable. The metric score includes values between 0 (most affluent) and 1 (most deprived). In addition, we changed the outcome variable and used linear regression modeling when examining the association between GISD and adherence to all three guidelines. In general, the results were in the same direction.

However, the modified model specifications clarified the earlier findings in two ways:

Regarding the relationship between GISD score and adherence to sleep guidelines, there was a significant effect in that adherence to the sleep duration guidelines increased with increasing deprivation (OR = 1.59 [1.04; 2.43], *p* = 0.031). Furthermore, the use of the GISD metric score also showed a significant relationship between GISD and PA levels in the sense that children and adolescents from more affluent neighborhoods were more likely to have higher levels of PA (b = −0.62 [−0.98; −0.26], *p* = 0.001). This was already indicated by the estimated margins in the logistic models (3.74 days of ≥60 min MVPA per week) (quintile 1) vs. 3.59 days of ≥60 min MVPA per week) (quintile 5)).

## 4. Discussion

In this study, we examined the association of regional socioeconomic deprivation with MVPA, ST, sleep behavior, and adherence to 24-h movement guidelines in a large sample of German children and adolescents (N = 17,433). We found a consistent association between deprivation and ST, with higher regional socioeconomic deprivation associated with more ST and lower odds of meeting ST guidelines. This appears to be a robust association, as it was found even after controlling for important covariates, including school type and perceived social status as SES-related variables. There were mixed results for PA and sleep duration or adherence to sleep duration guidelines. Students from the most affluent SES communities were more likely to meet all 24-h movement guidelines than those living in the most socioeconomically deprived areas. Overall, the proportion of adolescents meeting the MVPA, ST, and sleep duration guidelines was 14%, 22%, and 34%, respectively. Approximately 2% adhered to all three guidelines.

The vast majority of adolescents do not meet all of the three 24-h movement guidelines. In the current study, 2.3% of students met all three recommendations, consistent with the estimated 2.68% (95%CI: 1.78–3.58%) for adolescents recently reported in a meta-analysis [6]. The results indicate an alarmingly unhealthy lifestyle among today’s youth. Several health problems, such as cardiovascular and mental health problems, are associated with insufficient physical activity [2]. Excessive screen time in children and adolescents has been linked to obesity and mental health problems, for example [3,4]. The same has been found for poor sleep habits [5].

With our findings for ST, we replicated the results of other studies that examined the association between regional socioeconomic environment and ST. These studies often used neighborhood SES. A study from the Netherlands showed that a higher concentration of affluent neighbors, i.e., a lower level of deprivation, was associated with less ST in a sample of preadolescents [29]. Another study from Germany reported that lower neighborhood SES was associated with higher ST, while the association became insignificant after adjusting for school type and school neighborhood SES in 12- and 13-year-old students [21]. Our results complement these findings because we 1) examined a larger sample of children and adolescents aged 9–17 years from all over Germany and 2) examined SES at a more regional level than neighborhoods. We found a consistent relationship between regional socioeconomic deprivation, even after controlling for school type and perceived social status. Overall, the socioeconomic situation of regions or neighborhoods seems to play an important role in ST in youth. This may be due to a lack of access to alternative forms of entertainment or a greater reliance on electronic devices for communication and social connection in more deprived areas. Higher levels of education as an aspect of lower regional socioeconomic deprivation may also be associated with norms promoting healthier lifestyles [30]. The findings highlight the need for regional and community-based policy efforts and interventions to address excessive ST among children and adolescents in more deprived regions. In addition, ST may even be an important mediator of the reported association between area socioeconomic position and obesity in young people [20,31], which requires further investigation.

Our findings on PA reflect the inconsistent research on the influence of regional socioeconomic markers on PA in children and adolescents. While one study found that children living in low SES neighborhoods had lower levels of PA than those living in higher SES neighborhoods [32], another study found no association between the concentration of affluent neighbors and PA in a preadolescent sample [29]. Other regional characteristics, such as better neighborhood access to parks and playgrounds and social connectedness, were found to be important for PA [33]. There were even reverse effects in a Scottish sample of children, where children in more deprived areas had higher levels of out-of-school PA than those in less deprived areas [34]. Thus, the role of regional SES as a predictor of PA in children and adolescents is controversial. Furthermore, research on the relationship between SES and PA is further complicated by the multifaceted nature of SES and the lack of standardized definitions and measures [35]. While students in the most affluent quintile reported significantly more days with at least 60 min of MVPA in the unadjusted model, the associations were no longer significant after adjusting for school type and perceived social status as other indicators of SES. Similar results were found in another German sample [21]. These mixed findings highlight the need for further investigation of the role of different aspects of SES at individual to more regional levels and how these are interrelated.

Recent research on sleep behavior and SES suggests an association between lower SES levels and poorer sleep outcomes in terms of more symptoms of sleep disturbance and lower sleep satisfaction at lower SES levels. The effects of SES on sleep duration were inconsistent and dependent on the particular SES variable [36]. Higher perceived economic well-being and higher income were significantly associated with better sleep efficiency and longer sleep duration [37]. Our findings on sleep behavior do not fully reflect the current state of research. We found an inverse effect, with students from the most affluent areas reporting slightly shorter sleep duration than those from the most deprived regions. This effect in students has been found by others as well [38,39], while another meta-analysis found that poorer neighborhood SES was associated with shorter sleep duration in children (odds ratio: 1.26; 95% confidence interval: 1.086–1.467) [22]. The reason for this association may be that people with lower SES are more likely to live in environments that preclude optimal sleep [40]. In contrast to others, we found no association between regional socioeconomic deprivation and adherence to sleep guidelines. Based on current knowledge, people with low SES are likelier to be nonadherent than adherent [41]. Overall, the socioeconomic situation of a region or neighborhood appears to play a role in sleep duration and thus in adherence to sleep guidelines, but results to date are inconsistent.

Regarding sleep problems, our results reflect the current state of research on the influence of regional SES, where poorer sleep quality is associated with lower regional SES [36,42,43,44]. The findings are consistent with the effect found for increased ST in children and adolescents from deprived areas. In a review of ST and sleep in children and adolescents in Western countries, there was evidence of associations between ST and social media use with problems falling asleep and poor sleep quality in 13–15 year olds [45].

In summary, regional SES seems to be an important factor in the health behavior of children and adolescents in Germany. This is in contradiction to Article 72 of the Basic Law of the Federal Republic of Germany, which states as an important goal the establishment of equal living conditions throughout Germany. The results of this study show that this goal has not yet been achieved and that regional socioeconomic differences in Germany promote inequalities in health behaviors, especially concerning ST. Understanding these inequalities is important for developing effective interventions to promote healthy behaviors among adolescents, especially in low SES regions. First and foremost, measures should be taken to make parents and adolescents aware of increased media consumption and, in particular, its negative health effects. By identifying key factors that contribute to unhealthy behaviors in these communities, policymakers and health professionals can develop targeted interventions that address the unique needs and challenges of each region.

### 4.1. Strengths and Weaknesses of the Study

We included a large, heterogeneous sample recruited in 13 out of 16 German states, which can be considered a strength of our study. Another strength of the study is the use of GISD, an index of eight regional objective socioeconomic indicators of income, education, and occupation developed by the Robert Koch Institute, the German government’s central scientific institution in the field of biomedicine. We also controlled for the individual self-reported SES to clearly identify the association between the neighborhood SES and outcome variables. In addition to its strengths, however, the study also has weaknesses. The cross-sectional nature of the study is a major limitation. Therefore, no causal conclusions can be drawn. This must be taken into account when interpreting the results of the study. Another limitation is the self-reporting of health behaviors. In addition to difficulties in remembering and summing time intervals for different movement behaviors, response biases, such as social desirability, may have influenced the answers given. Finally, the GISD values were only available at the school level, not for each respondent’s place of residence. Therefore, we could not estimate social inequalities for each participant but only for the student body of a school.

### 4.2. Future Studies

Healthy lifestyle interventions should be developed, tested, and evaluated at the neighborhood level to reduce health disparities resulting from differences in regional social status. An initial concrete starting point is to reduce ST in disadvantaged neighborhoods. Parents and guardians in disadvantaged areas should be specifically educated about limiting ST in children and adolescents. Elementary schools in disadvantaged areas should include prevention programs to avoid excessive media use in their curricula, starting in the lower grades, and emphasize the health risks of excessive media use. Youth centers and drop-in centers for young people should also raise awareness and offer alternative ways to spend their free time.

## Figures and Tables

**Table 1:** Descriptive characteristics of the analysis samples.

	Total Sample	Adherence to Physical Activity Guidelines		Adherence to Screen Time Guidelines		Adherence to Sleep Time Guidelines		Adherence to All Guidelines	
		No	Yes	No	Yes	No	Yes	No	Yes
n	17,433 (100.0%)	14,150 (86.0%)	2204 (14.0%)	13,273 (78.0%)	3847 (22.0%)	10,912 (65.8%)	5405 (34.2%)	15,062 (97.7%)	338 (2.3%)
Mean age	13.1 (SD = 1.7)	13.2 (SD = 1.7)	12.9 (SD = 1.7)	13.4 (SD = 1.6)	12.0 (SD = 1.6)	13.3 (SD = 1.7)	12.8 (SD = 1.8)	13.1 (SD = 1.7)	12.0 (SD = 1.6)
Gender, female students	8685 (49.7%)	7297 (51.6%)	885 (40.2%)	6499 (49.0%)	2017 (52.4%)	5672 (52.0%)	2500 (50.1%)	7586 (50.4%)	134 (39.8%)
School type, students attending a Gymnasium	10,771 (60.4%)	8863 (62.6%)	1291 (58.5%)	7703 (58.0%)	2681 (69.6%)	6795 (62.2%)	3294 (60.9%)	9517 (63.2%)	228 (67.5%)
Perceived social status 1–10	7.1 (SD = 1.5)	7.0 (SD = 1.5)	7.3 (SD = 1.6)	7.0 (SD = 1.5)	7.2 (SD = 1.5)	7.0 (SD = 1.5)	7.2 (SD = 1.5)	7.0 (SD = 1.5)	7.7 (SD = 1.4)

SD, Standard Deviation.

**Table 2:** Results of logistic regression analyses for regional socioeconomic deprivation predicting adherence to 24-h movement guidelines.

	Meeting physical Activity Guidelines		Meeting Screen Time Guidelines		Meeting Sleep Time Guidelines		Meeting All Guidelines	
	OR 95% CI	*p*-Value	OR 95% CI	*p*-Value	OR 95% CI	*p*-Value	OR 95% CI	*p*-Value
Crude models								
GISD (Reference Group Quintile 1 (most affluent)								
2	1.01 0.79–1.28	0.961	0.67 0.44–1.00	0.049	0.95 0.72–1.25	0.691	0.69 0.38–1.27	0.234
3	0.95 0.74–1.23	0.705	0.49 0.32–0.74	0.001	1.08 0.82–1.43	0.589	0.64 0.33–1.23	0.177
4	0.86 0.68–1.09	0.204	0.48 0.32–0.71	<0.001	1.00 0.76–1.32	0.979	0.57 0.31–1.04	0.069
5 (most deprived)	0.94 0.74–1.19	0.608	0.44 0.30–0.66	<0.001	1.12 0.85–1.48	0.407	0.51 0.27–0.94	0.032
Adjusted models *								
GISD (Reference Group Quintile 1 (most affluent)								
2	0.86 0.70–1.12	0.295	0.78 0.60–1.01	0.055	0.90 0.70–1.15	0.389	0.70 0.41–1.19	0.185
3	0.86 0.67–1.11	0.245	0.64 0.48–0.84	0.001	1.06 0.82–1.37	0.656	0.71 0.40–1.26	0.238
4	0.80 0.64–1.01	0.058	0.54 0.42–0.69	<0.001	0.98 0.77–1.25	0.880	0.60 0.36–1.01	0.057
5 (most deprived)	0.87 0.69–1.10	0.239	0.49 0.38–0.64	<0.001	1.09 0.86–1.39	0.477	0.49 0.28–0.84	0.010

* Adjusted for age, gender, perceived social status, and school type. GISD German Index of socioeconomic deprivation, OR odds ratio, CI Confidence Interval.

**Table 3:** Results of linear regression analyses for regional socioeconomic deprivation predicting physical activity level, screen time, sleep time, and sleep problems.

	Physical Activity (Days with ≥60 Min MVPA per Week)		Screen Time (h/Day)		Sleep Time (h/Night)		Sleep Problems (Frequency)	
	b 95% CI	*p*-Value	b 95% CI	*p*-Value	b 95% CI	*p*-Value	b 95% CI	*p*-Value
Crude models								
GISD (Reference Group Quintile 1 (most affluent)								
2	−0.07 −0.27–0.13	0.489	0.50 0.16–0.83	0.004	0.04 −0.18–0.27	0.697	0.15 0.04–0.27	0.009
3	−0.21 −0.43–−0.01	0.058	0.84 0.49–1.19	<0.001	0.09 −0.14–0.33	0.435	0.20 0.07–0.32	0.002
4	−0.16 −0.36–0.04	0.127	0.88 0.56–1.20	<0.001	0.06 −0.16–0.28	0.583	0.16 0.05–0.28	0.005
5 (most deprived)	−0.21 −0.42–−0.01	0.042	0.81 0.48–1.14	<0.001	0.18 −0.04–0.41	0.110	0.23 0.11–0.35	<0.001
Adjusted models *								
GISD (Reference Group Quintile 1 (most affluent)								
2	−0.01 −0.18–0.20	0.925	0.23 0.04–0.45	0.047	−0.03 −0.15–0.09	0.621	0.10 −0.01–0.21	0.053
3	−0.09 −0.29–0.11	0.394	0.46 0.22–0.70	<0.001	0.09 −0.04–0.21	0.190	0.13 0.01–0.25	0.035
4	−0.10 −0.28–0.09	0.299	0.69 0.48–0.90	<0.001	0.03 −0.09–0.14	0.646	0.14 0.04–0.24	0.005
5 (most deprived)	−0.15 −0.34–0.04	0.131	0.61 0.40–0.83	<0.001	0.13 0.02–0.25	0.026	0.19 0.08–0.29	0.001

* Adjusted for age, gender, perceived social status, and school type. GISD, German Index of socioeconomic deprivation; b, regression coefficient; CI, Confidence Interval.

## Data Availability

The data that support the findings of this study are available from the corresponding author upon reasonable request.
